# Marker-assisted forward and backcross breeding for improvement of elite Indian rice variety Naveen for multiple biotic and abiotic stress tolerance

**DOI:** 10.1371/journal.pone.0256721

**Published:** 2021-09-02

**Authors:** Perumalla Janaki Ramayya, Vishnu Prasanth Vinukonda, Uma Maheshwar Singh, Shamshad Alam, Challa Venkateshwarlu, Abhilash Kumar Vipparla, Shilpi Dixit, Shailesh Yadav, Ragavendran Abbai, Jyothi Badri, Ram T., Ayyagari Phani Padmakumari, Vikas Kumar Singh, Arvind Kumar

**Affiliations:** 1 International Rice Research Institute (IRRI), South-Asia Hub, ICRISAT, Hyderabad, India; 2 International Rice Research Institute, South Asia Regional Centre (ISARC), Varanasi, India; 3 Leibniz Institute of Plant Genetics and Crop Plant Research (IPK), Gatersleben, Germany; 4 ICAR-Indian Institute of Rice Research (IIRR), Rajendra Nagar, Hyderabad, India; Faculty of Agriculture (FoA), Sher-e-Kashmir University of Agricultural Sciences and Technology of Kashmir (SKUAST-K), Wadura Campus, INDIA

## Abstract

The elite Indian rice variety, Naveen is highly susceptible to major biotic and abiotic stresses such as blast, bacterial blight (BB), gall midge (GM) and drought which limit its productivity in rainfed areas. In the present study, a combined approach of marker-assisted forward (MAFB) and back cross (MABC) breeding was followed to introgress three major genes, viz., *Pi9* for blast, *Xa21* for bacterial blight (BB), and *Gm8* for gall midge (GM) and three major QTLs, *viz*., *qDTY*_*1*.*1*_, *qDTY*_*2*.*2*_ and *qDTY*_*4*.*1*_ conferring increased yield under drought in the background of Naveen. At each stage of advancement, gene-based/linked markers were used for the foreground selection of biotic and abiotic stress tolerant genes/QTLs. Intensive phenotype-based selections were performed in the field for identification of lines with high level of resistance against blast, BB, GM and drought tolerance without yield penalty under non-stress situation. A set of 8 MAFB lines and 12 MABC lines with 3 to 6 genes/QTLs and possessing resistance/tolerance against biotic stresses and reproductive stage drought stress with better yield performance compared to Naveen were developed. Lines developed through combined MAFB and MABC performed better than lines developed only through MAFB. This study exemplifies the utility of the combined approach of marker-assisted forward and backcrosses breeding for targeted improvement of multiple biotic and abiotic stress resistance in the background of popular mega varieties.

## Introduction

Rice (*Oryza sativa* L.) is the principal food crop of the world. More than one-third of the world’s population and half of India’s population relied on rice as their primary calorie intake. It is estimated that, about 135–140 million tons of rice needs to be produced in India by 2025 to keep pace with the increasing population [1]. The decrease in the area of cultivable land, water scarcity, emergence of new diseases and pests and change in climatic conditions causing a serious threat to rice production. The ever-changing climatic factors found to expand the host range of pathogens with increased chances of the occurrence of simultaneous stresses on the plant. Rice varieties that can tolerate independent stresses may not necessarily tolerate the simultaneous occurrence of two or more stresses [2, 3]. Therefore, there is a need for the development of high yielding and multiple stress tolerant rice varieties

Blast, Bacterial blight (BB), gall midge (GM), and drought are the major biotic and abiotic stresses in rice leading to significant yield losses in the majority of the rainfed rice-growing regions. To combat both biotic and abiotic stresses simultaneously, the development and cultivation of resistant/tolerant rice varieties could be the most economical, effective and environment friendly approach. So far, none of the genes that can confer resistance/tolerance to multiple biotic and abiotic stresses concurrently have been identified. Hence, pyramiding multiple genes/QTL together into a variety helps in the development of lines possessing tolerance/resistance to multiple biotic and abiotic stresses. Availability of DNA based genetic markers for the resistance associated genes/QTLs facilitates their transfer into high yielding rice varieties through marker-assisted selection. In rice, blast is one of the most destructive diseases caused by *Magnaporthe oryzae* fungi. It occurs in the majority of the rice-cultivating areas throughout the world and results in as high as 70–80% yield loss [4]. To date, about 100 distinct blast resistance genes and 350 QTLs have been identified. Nearly 30 genes were cloned from these [5]. Among the blast R genes in India, *Pi1*, *Pi2*, *Pi9 and Pi54* are most effective against a wide range of fungal isolates [6]. *Pi9* is a major blast resistant gene identified from the wild species *Oryza minuta* which provides broad spectrum resistance against diverse *M*. *oryzae* isolates [7–9]. Introgression of *Pi9* gene into Pusa Basmati 1 resulted in higher resistance to blast and the improved variety Pusa 1637-18-7-6-20 now growing in larger area compared to Pusa Basmati 1 [10].

Bacterial blight (BB) is caused by *Xanthomonas oryzae* pv. oryzae and there are about 22 pathotypes identified from diverse geographical locations across India. So far, nearly 45 BB resistance genes have been identified [11], among them, nine were cloned (*Xa1*, *xa5*, *xa10*, *xa13*, *Xa21*, *Xa23*, *Xa25*, and *Xa27* [12, 13] and seven genes (*Xa4*, *Xa7*, *Xa22*, *Xa30*, *Xa31*, *Xa33* and *Xa34*) were fine mapped. Among the identified resistance genes, three genes (*xa5*, *xa13* and *Xa21*) are found to be highly effective in Indian context and incorporated successfully into high yielding elite rice varieties [13–18].

Gall midge (*Orseolia oryzae)* is a serious pest causing significant crop damage in some regions of the east, central and south India, mostly during wet season. An annual yield loss of about 0.8% of the total production is observed in India [19]. To date, 11 gall midge resistance genes have been identified in rice, among them eight genes (*Gm1*, *Gm2*, *Gm4*, *Gm5*, *Gm6*, *Gm7*, *Gm8* and *Gm11*) have been mapped [20–23] and four candidate genes *Gm2*, *gm3*, *Gm4* and *Gm8* have been cloned and characterized [24]. Seven biotypes of the pest have been reported in India so far and the resistance genes *Gm1*, *gm3*, *Gm4*, and *Gm8* are found to be resistant against all the 7 biotypes [25, 26]. The resistant genes *Gm8*, *Gm1* and *Gm4* were successfully introgressed into RPHR-1005 [27] and elite variety improved Lalat [28] through marker-assisted backcross breeding (MABC).

Drought is the major abiotic stress that occurs in rice as its cultivation requires a high amount of water. According to climate change reports, it is estimated that there will be more water deficit across South Asia and Southeast Asia in the years to come. In rainfed areas, water stress is one of the most serious problems that can occur at any time during the growth period of rice. The reproductive stage in rice is more sensitive to water stress which negatively affects the growth and yield potential of the plant [16]. Most of the popular high yielding rice varieties grown in rainfed areas is highly sensitive to drought stress. There is an urgent need for the development of tolerant rice varieties which can withstand water deficit conditions. In the last few years, several QTLs (*qDTYs*) has been identified for yield under drought stress at IRRI [29–37] and successfully introgressed into high yielding rice varieties which are susceptible to drought through marker-assisted selection [33, 34, 38, 39]. Among the 12 major drought yield under stress QTLs, seven QTLs (*qDTY*_*1*.*1*_, *qDTY*_*2*.*2*_, *qDTY*_*3*.*1*_, *qDTY*_*3*.*2*_, *qDTY*_*4*.*1*_, *qDTY*_*6*.*1*_, and *qDTY*_*12*.*1*_*)* were reported with large effects across different genetic backgrounds under both transplanted lowland and direct seeded upland environments. Through introgression of two QTLs (*qDTY*_*2*.*2*_ and *qDTY*_*4*.*1*_ into popular high-yielding rice variety IR64, yield advantage of 0.8–1.0 t/ha was reported under severe drought stress [40].

The aim of the present study is to introgress blast, BB, GM resistance genes and drought-tolerant QTLs into popular high yielding rice variety Naveen and also to assemble these genes/QTLs to develop lines with increased resistance/tolerance against multiple biotic and abiotic stresses. Rice variety Naveen was released for cultivation in the states of Odisha (2006), Tripura (2012) of India. It was developed by crossing early duration upland variety, Sattari with the popular high yielding rice variety, Jaya. The average productivity of Naveen is estimated to be 5.0–6.0 t/ha. It is a semi-dwarf (105 cm) rice variety with mid-early duration (115–120 days) and medium-bold grain type [41]. Though Naveen is having high popularity, but it is highly susceptible to blast, BB, and drought. In the present study, attempts were made to simultaneously improve the Naveen for resistance/tolerance to both biotic and abiotic stress through marker-assisted forward (MAFB) and backcrossing (MABC) of stress resistance genes.

## Materials and methods

### Plant material and breeding strategy

**Fig 1:**
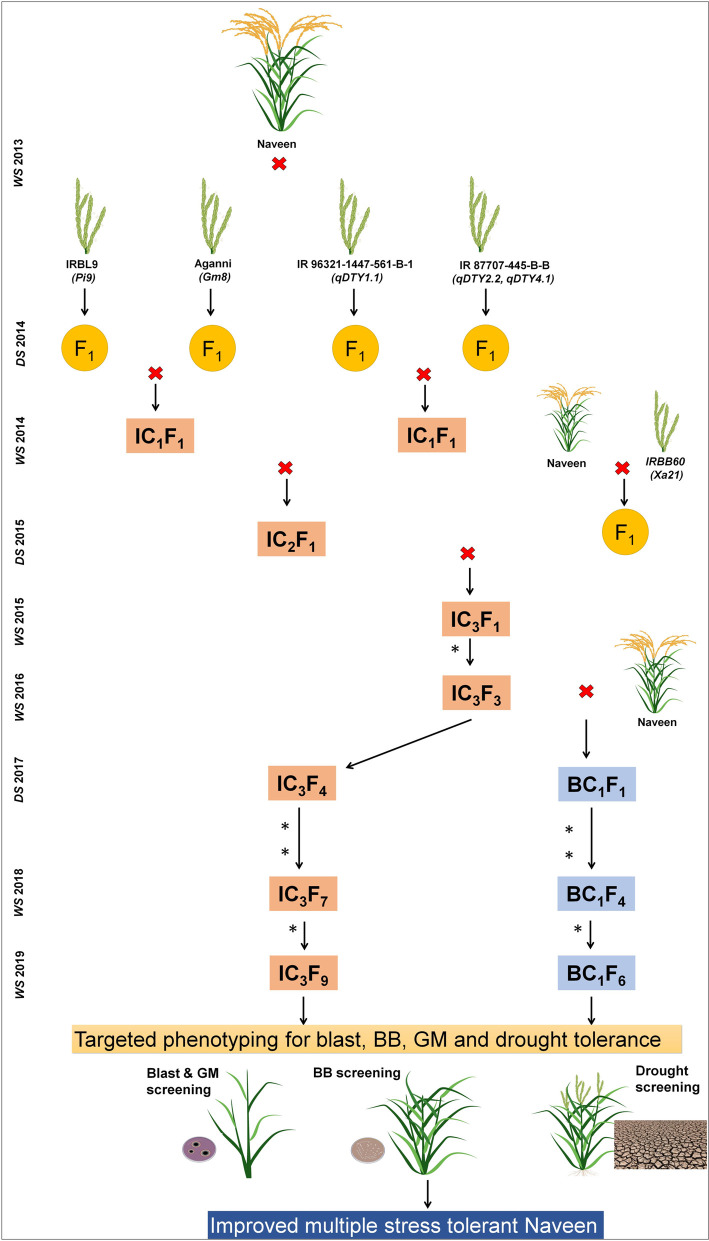
Flow diagram depicting the different steps involved in development of gene/QTL pyramids of Naveen through Marker-Assisted Forward Breeding (MAFB) and Marker-Assisted Backcross Breeding (MABC). *Generation advancement by selfing.

The experiments in the present study were carried out at International Rice Research Institute (IRRI), South Asia Hub located at ICRISAT, Patancheru. ICRISAT Research farm is situated at an altitude of 545 meter above mean sea level with latitude of 17.53°N and longitude of 78.27°E. The breeding lines, IRBL9, possessing blast resistance gene (*Pi9*), IRBB60 possessing four BB resistance gene (*Xa4*, *xa5*, *xa13* and *Xa21*, however we have targeted only *Xa21* gene), and Aganni possessing gall midge resistance gene (*Gm8*), IR 96321-1447-561-B-1 (*qDTY*_*1*.*1*_) and IR 87707-445-B (*qDTY*_*2*.*2*_ and *qDTY*_*4*.*1*_) possessing drought-tolerant QTLs were used as the donor parents to cross with recipient parent Naveen. During WS2013, four independent crosses were made between Naveen and different donor parents (IRBL9, Aganni, IR 96321-1447-561-B-1 and IR 87707-445-B) (Fig 1). The resultant true F_1_s were intercrossed during DS2014 to produce intercross F_1_s (IC_1_F_1_) plants. Meanwhile, another independent cross was made between IRBB60 and Naveen to introgress *Xa21* gene into Naveen. At each cross, F_1_s were genotyped with trait linked markers and plants containing the alleles of donor parents were selected at the IC_1_F_1_ generation. During WS2014, true intercrossed F_1_s (IC_1_F_1_) was again crossed with each other to produce IC_2_F_1_s. During DS2015, the true IC_2_F_1_s were crossed with the true F_1_ of Naveen/IRBB60 and produced IC_3_F_1_s. The IC_3_F_1_ plants were confirmed genotypically for the presence of tolerant/resistance genes and the lines with maximum genes/QTLs combinations and better phenotypic expression were forwarded to IC_3_F_3._ Superior plants in the IC_3_F_3_ generation with maximum number of genes/QTLs combinations were selected and selfed up to IC_3_F_7_ as MAFB. In parallel, MABC program was initiated and the superior plants of IC_3_F_3_ generation with all the genes/QTLs combinations were backcrossed with recurrent parent Naveen to produce BC_1_F_1s._ The MABC lines were selfed up to BC_1_F_4_. These lines were phenotypically tested for two consecutive seasons for their resistance to blast, BB, one season for GM resistance and at two locations for drought tolerance. The detailed breeding strategy is presented in Fig 1.

### Parental polymorphism and genotyping

A parental polymorphism survey was conducted with the recurrent parent Naveen and the donor parents IRBL9, IRBB60, Aganni, IR 96321-1447-561-B-1 and IR 87707-445-B using the marker information collected from literature for all the 3 targeted genes and 3 drought QTLs. The purpose is to examine the relationship between the markers and the resistance/tolerance and also susceptible nature of the parents.

Due to the large population size and the number of markers, a technically simple, quick, and reproducible DNA isolation technique was carried out according to the International Rice Research Institute (IRRI) protocol (TPS buffer) for genotyping [42]. In each generation, fresh DNA was isolated from the parents and their respective progenies 15 days after transplantation to check the presence of desirable genes/QTLs. For blast resistance, *Pi9* gene, the tightly linked marker *Pi9STS2* [7], for BB resistance, the *Xa21* gene, the tightly linked SSR marker *pTA248* [43] and for GM resistance, *Gm8* tightly linked marker *GM8 PRP* was used for foreground selection at each generation. For the drought tolerance QTLs, SSR markers RM3825, RM431 and RM12091 used for *qDTY*_*1*.*1*_ [31], RM154, RM279, RM555 used for *qDTY*_*2*.*2*_ and RM551, RM518 and 16367 used for *qDTY*_*4*.*1*_ [40] respectively. The information of the genes/QTLs, tightly linked markers and their sequences were presented in S1 Table.

PCR reaction was performed in a total volume of 10 μl containing 50 ng of DNA template, 1 μl 10X PCR buffer, 2.5 pico M of each forward and reverse primer, 75 μM of each dNTP, and 0.5U of Taq DNA polymerase (Geneilabs, India). The PCR amplification cycle was performed based on standardized annealing temperatures specific to each marker representing gene/QTL. Products were resolved in a 3.0% agarose gel stained with EtBr and the gel images were documented in SYNGENE imager (SYNGENE, USA).

### Phenotypic screening for blast resistance

All the pyramided lines (F_7,_ F_9,_ and BC_1_F_4_, BC_1_F_6_) along with donor (IRBL9), recipient (Naveen) and susceptible checks (HR-12) were evaluated in uniform blast nursery (UBN) located at IRRI- South Asia Hub (IRRI-SAH), Hyderabad using a highly virulent isolate collected from Andhra Pradesh, India. The screening was done during WS2018 and WS2019. A 50-cm-long row of each entry was raised in nursery beds with a row spacing of 10 cm. Between every four entries and also along with borders, one row of the susceptible check (HR-12) was planted to facilitate the uniform spread of the disease. The inoculum with the concentrations 1 x 10^5^spore/ml was sprayed onto young seedlings at four-leaf stages using a fine sprayer and high relative humidity was maintained for disease development. Disease reaction of each line was recorded on 30 days after sowing and continued at 5 days intervals until the susceptible check had more than 80% of infection. Disease reaction was scored visually on 0–9 SES scale according to Standard Evaluation System, IRRI [44]. SES scores 0–3 were considered resistant (R), 4–5 as moderately resistant (MR) and 6–9 as susceptible.

### Phenotypic screening for bacterial leaf blight resistance

A highly virulent isolate of bacterial blight pathogen, *Xanthomonas oryzae* pv. oryzae (*Xoo*) IX-020 collected from Hyderabad, Telangana [45], were used to screen the pyramided lines (F_7,_ F_9_ and BC_1_F_4_, BC_1_F_6_) along with donor (IRBB60), recipient (Naveen) and susceptible check (TN1). The artificial clip inoculation method was followed for inoculation in field conditions. The *Xoo* strains were cultured and multiplied on peptone sucrose agar media for inoculums production. The rice plants were inoculated at maximum tillering stage (50–55 days) with a bacterial suspension of 10^9^cfu/ml following clip inoculation method as described by [46]. A total of 12–15 fully expanded leaves from four plants of each line were clip inoculated. The disease reaction was scored on the inoculated plants after 21 days of inoculation according to the IRRI standard evaluation system (SES) by measuring the lesion length from the cut tip of inoculated leaves [44]. A plant was classified as resistant if the average lesion length was shorter than 3 cm, moderately resistant if the lesion was >3–6 cm, moderately susceptible if the lesion was >6–9 cm, and susceptible if the lesion was longer than >9 cm [47].

### Phenotypic screening for gall midge resistance

Screening for gall midge resistance was conducted in a greenhouse at ICAR-IIRR, Hyderabad according to standard procedures [48]. Pre-germinated seeds of selected pyramided lines, donor parent and a susceptible check (TN1) were planted in seed boxes (60 x 45 x 10 cm) in rows with 5 cm distance between rows and 10–15 plants per row. After 12 days of sowing, 30 female and 15 male insects of gall midge biotype 1 (GMB1) were released onto the seedlings for 2 consecutive days and the trays were covered with nylon mesh to prevent the escape of adults. The trays were placed in a humid chamber with 90% relative humidity (RH) and at 30 ± 5°C temperature for egg incubation and larval establishment. After two days, the trays were shifted to growth chambers and maintained at room temperatures (25 ± 10°C and 60 ± 10% RH) for maggot development. Disease scores like seedling damage (in %), emergence and frequency of silver shoot (galls) were recorded after twenty days when the susceptible check, TN1 showed fully extended galls (90–100% damage). Plants were scored individually based on standard scoring system proposed by the International Rice Research Institute [44]

### Phenotypic screening for drought tolerance and agronomic performance

All the pyramided lines (F_7_ and BC_1_F_4_) along with recurrent and donor parents and susceptible checks were screened for reproductive stage drought tolerance during WS2018 at two test fields (IRRI-SAH, Hyderabad and ICAR-IIRR, Hyderabad) as described by [49]. Twenty one-day-old seedlings of all the lines along with respective parents were transplanted in the field in an augmented randomized complete block design (Aug-RCBD) with repeated checks and a plot size of 2.4 m^2^ for the drought stress and non-stress experiment. The row-to-row and hill -to- hill spacing of 20 cm and 15 cm respectively in the lowland were maintained. Standard agronomic practices were followed while growing the rice plants. Irrigation was supplied continuously in a non-stress experiment until maturity. In stress experiment, irrigation was stopped 30 days after transplantation and the remaining water was drained out from the field to initiate stress. Moisture content in the below ground soil was monitored by inserting 100 cm perforated PVC pipes into the soil in stress experimental field in a zig-zag manner. The decline in water table depth was measured on a daily basis with a meter scale inserted into the PVC pipes. One life-saving irrigation was provided for 24h when water table level reached 100 cm below the soil surface and most lines were wilted and exhibited severe leaf drying. Then, a second cycle of the stress was initiated which continued till maturity.

Along with grain yield under drought, the pyramided lines (F_7_ and BC_1_F_4_) were evaluated for various agronomic traits at both the test field (IRRI-SAH and ICAR-IIRR). Extensive phenotypic selection for the advancement of introgressed segregating material during every generation was carried out after the genotypic confirmation of plants at initial stage. Plants with maximum positive genes were tagged in the field and their phenotypic performance was evaluated. Phenotyping data collection involved days to 50% flowering (DTF), plant height (PH), number of productive tillers per plant (PT), panicle length (PL), grain yield per plant (GY kg ha^-1^) and 1000 grain weight (TGW) according to [50]. Harvesting was done at physiological maturity after which GY was measured per plot. Seeds were dried to 12% moisture before weighing and the plot yield was converted to kg ha^–1^. Descriptive statistics was performed using PB tools for the phenotypic traits (DTF, PH, PT, PL, GY and TGW) recorded for MAFB and MABC lines from both the test fields.

### Evaluation of grain quality

Grain quality analysis was performed for all the pyramided lines (MAFB and MABC) during WS2018. After harvest, the rice grains were dried under direct sunlight up to 14% moisture content. Twenty grains from each entry were dehusked, polished and evaluated for physical characters like kernel length, kernel width and L/B ratio using a digital Vernier caliper. The cooking quality traits such as, Alkali spread value (ASV), gel consistency (GC) and amylose content (%) were estimated according to [51].

## Results

### Marker-assisted forward breeding for pyramiding multiple genes/QTLs

The marker-assisted forward breeding approach was followed in the present study to transfer six genes/QTLs to complement the blast, BB, GM resistance and drought tolerance in elite rice variety Naveen. Initially, during WS2013 Naveen was crossed with all four different donors individually and F_1_ progeny developed. Single crosses of Naveen with IRBL9 (*Pi9*) and Aganni (*Gm8*) resulted in 150 and 112 F_1_s. Foreground selection with *Pi9*STS-2 and *PRP* resulted in the identification of 103 and 91 true F_1_s with the presence of *Pi9* and *Gm8* genes respectively. Likewise, separate crossings attempted between Naveen with IR 96321-1447-561-B-1(*qDTY*_*1*.*1*_) and Naveen with IR 87707-445-B-B (*qDTY*_*2*.*2*_ and *qDTY*_*4*.*1*_) resulted in development of 85 and 130 F_1_s, respectively. Foreground selection with *qDTY*_*1*.*1*_ specific co-dominant markers RM3825 (right flaking marker), RM431 (peak marker) and RM12091 (left flanking marker), *qDTY*_*2*.*2*_ specific markers RM154 (right flaking marker), RM279 (left flanking marker) and *qDTY*_*4*.*1*_ specific markers RM551 (right flaking marker), RM518 (peak marker) and RM16367 (left flanking marker) resulted in identification of 76 and 54 true F_1_s possessing *qDTY*_*1*.*1*_ and *qDTY*_*2*.*2*_, *qDTY*_*4*.*1*_.

All the true F_1_s were grown in the field during DS2014 and intercrosses were made among the true F_1_s_,_ one plant from each of the crosses were selected to develop IC_1_F_1_s for pyramiding the targeted multiple gene/QTLs. The heterozygous F_1_s of Naveen/IRBL9 crossed with heterozygous F_1_s of Naveen/Aganni to pyramid blast (*Pi9*) and gall midge (*Gm8*) genes into Naveen background resulted in the generation of 163 plants, out of which the 37 plants were identified as true IC_1_F_1_s with both *Pi9* and *Gm8* genes using foreground selection. Similarly, the heterozygous F_1_ plant of Naveen/IR 96321-1447-561-B-1 crossed with a heterozygous F_1_ plant of Naveen/IR 87707-445-B-B to pyramid all three drought-tolerant QTLs into Naveen background resulted in development of total of 302 IC_1_F_1_s and foreground selection with *qDTY* specific markers resulted in confirmation of 31 true IC_1_F_1_s with all three targeted *qDTYs* (*qDTY*_*1*.*1*_, *qDTY*_*2*.*2*_ and *qDTY*_*4*.*1*_). During WS2014, intercrosses were performed between IC_1_F_1_s possessing *Pi9*, *Gm8* genes and IC_1_F_1_s possessing three drought QTLs to bring all of them into the same background. This results in development of a total of 2935 IC_2_F_1_s, out of which 102 F_1_s with different gene/QTL combinations (1–5 gene/QTL) were identified through foreground selection. In the same season, Naveen was crossed with IRBB60 to introgress *Xa21* gene and identified 84 true F1s. During DS2015, the positive IC_2_F_1_ plants with all five gene/QTL combinations were intercrossed with true F_1_s from Naveen/IRBB60 to the pyramid *Xa21* (BB) gene and generated a total of 3015 IC_3_F_1_ plants. During WS2015, all the IC_3_F_1s_ were grown in the field and stepwise foreground selection was performed with all the markers for selection of lines possessing all 6 genes/QTLs combinations. This resulted in the identification of 35 positive plants with all the genes/QTLs but the majority of the lines with *Xa21* and *Pi9* genes were in heterozygous condition.

All positive plants for the desired traits of interest were selfed to produce F_2_s. During DS2016, a total of 6145 IC_3_F_2_ plants were developed among which 1663 IC_3_F_2_ plants were selected through extensive field selections and further used for genotyping with trait linked markers. Out of 1663 IC_3_F_2_ plants, 37 plants were found with different gene/QTL combinations which were forwarded to IC_3_F_3_. In WS2016, around 5000 IC_3_F_3_ seeds were generated from 37 IC_3_F_3_ as single plant and the selective genotyping of the plants during early stage resulted in selection of 15 positive plants with different gene/QTL combinations. Out of 15 plants, 8 plants were forwarded to IC_3_F_4_ and the remaining 7 plants were used for backcrossing. During DS2017, 2520 F_4_ seeds were obtained from 8 plants and were screened genotypically and phenotypically and 11 plants with different genes/QTLs combinations and better agronomic performance were forwarded to IC_3_F_5_. During WS2017, 1183 seeds were obtained from 11 plants, out of which 6 plants with different gene/QTL combinations were selected. During DS2018, a total of 1200 IC_3_F_6_ seeds were derived from 6 IC_3_F_5_ plants. Finally, through stringent phenotypic and genotypic screening, 8 plants were selected from IC_3_F_6_ possessing different genes/QTLs combination and providing better yield performance and were bulked for evaluating yield, biotic and drought stress tolerance.

### Marker-assisted backcross breeding for pyramiding multiple genes/QTLs into Naveen

In parallel to MAFB, the backcross breeding approach was also followed to develop the NILs with multiple stress tolerance. Superior plants from 7 IC_3_F_3_ plants progenies possessing all genes/QTLs combinations (*Xa21*, *Gm8*, *Pi9*, *qDTY*_*1*.*1*_, *qDTY*_*2*.*2*_ and *qDTY*_*4*.*1*_) and better phenotypic expression were selected from forward breeding program and were backcrossed with the recurrent parent Naveen during WS2016. A total of 1065 BC_1_F_1_ seeds were generated and grown in the field during DS2017. Foreground selection was performed with selected markers and 5 plants possessing all 6 genes/QTLs combinations identified. During WS2017, the 5 selected BC_1_F_1_ plants were selfed to produce 8400 seeds which were sown in the field. As it is very difficult to genotype all the 8400 plants, we have selected 250 plants from each BC_1_F_1_ plant through extensive field selections based on grain and plant type and finally selected 1250 BC_1_F_2_ seeds which were sown in field and genotyped with trait linked markers. From these 1250 plants, 5 superior plants were forwarded to BC_1_F_3_. In DS2018, a total of 882 BC_1_F_3_ seeds derived from 5 plants were screened in the field. Based on the genotypic analysis, 12 plants with different genes/QTLs combinations were selected and forwarded to BC_1_F_4_. These were selfed to produce BC_1_F_5_ and BC_1_F_6_ generations and bulked for evaluation for yield, biotic and drought stress tolerance along with forward breeding lines.

### Bioassay of the marker-assisted breeding lines for biotic stress resistance

**Fig 2:**
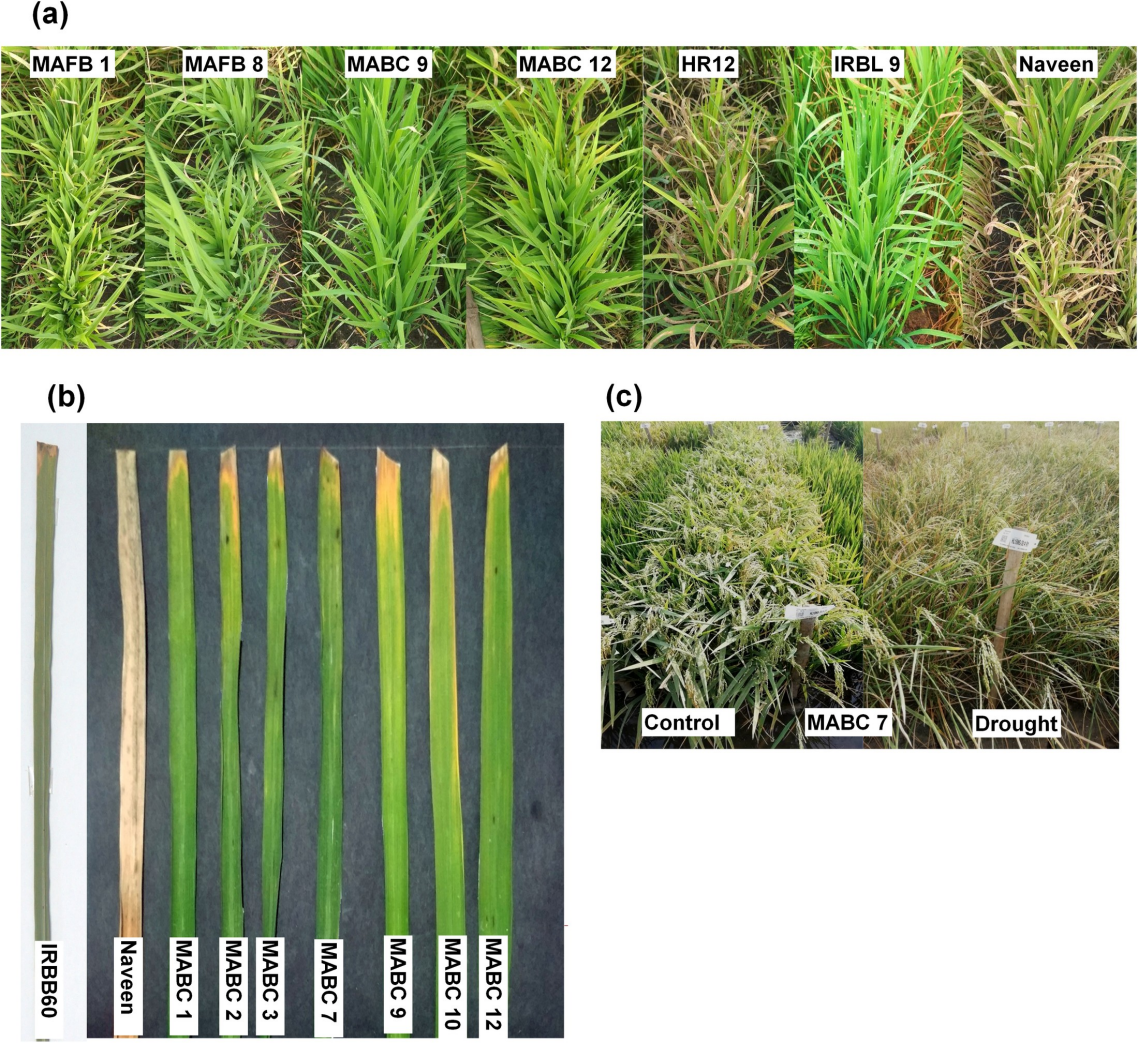
Representative picture of screening of the ILs for blast, BLB, and drought. (a) screening for blast resistance (b) screening for bacterial leaf blight resistance (c) Screening for drought tolerance.

Marker-assisted breeding lines developed through both MAFB and MABC approach were further evaluated for the targeted biotic stress traits *viz*., blast, BB and GM resistance. A set of 8 IC_3_F_7_ lines obtained through forward breeding approach containing 3 to 5 gene/QTLs combinations and a set of 12 BC_1_F_4_ lines with 3 to 6 genes/QTLs combinations were evaluated for blast and BB resistance for 2 consecutive seasons (WS2018 and WS2019) at IRRI-SAH, Hyderabad. Recurrent parent Naveen, donor IRBL9, susceptible checks HR12 was also included in the screening to study their reaction to the blast inoculation. In both the seasons, the donors IRBL9 showed resistant reaction (R) with a score of 0 while the recurrent parent and susceptible checks showed susceptible reaction with the score of 7. Out of 8 MAFB lines, only 2 lines (MAFB 1 and MAFB 8) found to possess *Pi9* gene and showed high degree of resistance to blast whereas MAFB 4 and MAFB 6 showed moderate resistance (MR) though there is no *Pi9* identified in these lines genotypically **(Table 1)**. Similarly among the 12 MABC lines, 3 lines (MABC 5, MABC 9 and MABC12) found to posses *Pi9* gene, among them 2 lines showed higher level of resistance **(Fig 2A)** and 1 line (MABC 5) showed susceptible reaction. Apart from this, 3 lines (MABC 2, MABC 8 and MABC 11) showed moderate resistance (MR) though there is no *Pi9 gene* **(Table 2)**.

**Table 1:** Disease reaction (blast, bacterial blight, and gall midge) of pyramided lines with different gene/QTL combinations developed through Marker Assisted Forwarding Breeding (MAFB) approach.

Gene/QTL pyramids	Blast Score		BB lesion length (cm)		Gall midge *	Gene/QTL combination	No. of genes
	WS2018	WS2019	WS2018	WS2019	WS2018		
MAFB 1	2 (R)	1 (R)	5 ± 0.4 (MR)	4 ± 0.3 (MR)	R	*Gm8+Pi9 +Xa21+qDTY*_*2*.*2*_ *+qDTY*_*4*.*1*_	5
MAFB 2	6 (S)	6 (S)	5 ± 0.3 (MR)	6 ± 0.6 (MR)	S	*Xa21+qDTY*_*1*.*1*_ *+qDTY*_*4*.*1*_	3
MAFB 3	6 (S)	6 (S)	12 ± 0.6 (S)	10 ± 0.7 (S)	R	*Gm8+qDTY*_*2*.*2*_ *+qDTY*_*4*.*1*_	3
MAFB 4	5 (MR)	5 (MR)	11 ± 0.3 (S)	14 ± 0.8 (S)	R	*Gm8+qDTY*_*1*.*1*_ *+qDTY*_*2*.*2*_ *+qDTY*_*4*.*1*_	4
MAFB 5	7 (S)	7 (S)	1 ± 0.5 (R)	3 ± 0.4 (R)	R	*Gm8 +Xa21 +qDTY*_*2*.*2*_ *+qDTY*_*4*.*1*_	4
MAFB 6	5 (MR)	5 (MR)	0 ± 0.2 (R)	3 ± 0.2 (R)	R	*Gm8+Xa21+qDTY*_*2*.*2*_ *+qDTY*_*4*.*1*_	4
MAFB 7	8 (S)	7 (S)	2 ± 0.5 (R)	2 ± 0.5 (R)	S	*Xa21+qDTY1*.*1+qDTY*_*2*.*2*_*+qDTY*_*4*.*1*_	4
MAFB 8	2 (R)	1 (R)	2 ± 0.4 (R)	2 ± 0.3 (R)	S	*Pi9+Xa21+qDTY*_*1*.*1*_ *+qDTY*_*2*.*2*_ *+qDTY*_*4*.*1*_	5
Naveen	7 (S)	7 (S)	13 ± 0.5 (S)	14.5 ± 0.4 (S)	S	-	-

Blast Resistant Check- IRBL9 (2), TETEP (0); Blast Susceptible Check- HR12 (7); BLB Resistant Check- IRBB60 (0.5 cm); BLB Susceptible Check- TN1 (19 ± 0.8); Gall midge resistant check–Aganni (0 galls); Gall midge susceptible check–TN1 (100 galls).

S-Susceptible; R-Resistance; MR-Moderate resistance.

* Screened at IIRR, Hyderabad “GM glass house screening facility” using Biotype 1 during WS2018

Blast (SES, IRRI 2013); 0–2 (Resistant), 3–4 (Moderately Resistant), 5–9 (Susceptible)

BLB lesion length Chen., et al. 2000 R (up to 3 cm), MR (> 3–6 cm), MS (> 6–9 cm), S (> 9 cm)

Gall Midge (SES, IRRI 2002); 0, 1, 3 Resistant and 5, 7, 9 Susceptible.

**Table 2:** Disease reaction (blast, bacterial blight and gall midge) of pyramided lines with different gene/QTL combinations developed through marker assisted backcross approach.

Gene/QTL pyramids	Blast Score		BB lesion length (cm)		Gall midge*	Gene/QTL combination	No. of genes
	WS2018	WS2019	WS2018	WS2019	WS2018		
MABC 1	7 (S)	8 (S)	3 ± 0.5 (R)	3 ± 0.5 (R)	R	*Gm8 +Xa21 +qDTY*_*1*.*1*_ *+qDTY*_*2*.*2*_ *+qDTY*_*4*.*1*_	5
MABC 2	4 (MR)	5 (MR)	0 ± 0 (R)	2 ± 0.3 (R)	R	*Gm8 +Xa21 +qDTY*_*1*.*1*_ *+qDTY*_*2*.*2*_ *+qDTY*_*4*.*1*_	5
MABC 3	6 (S)	7 (S)	0 ± 0.1 (R)	3 ± 0.3 (R)	R	*Gm8 +Xa21 +qDTY*_*1*.*1*_ *+qDTY*_*2*.*2*_ *+qDTY*_*4*.*1*_	5
MABC 4	7 (S)	7 (S)	4 ± 0.6 (MR)	4 ± 0.4 (MR)	R	*Gm8 +Xa21 +qDTY*_*1*.*1*_	3
MABC 5	6 (S)	6 (S)	9 ± 0.5 (S)	9 ± 0.5 (S)	R	*Gm8 +Pi9 +Xa21 +qDTY*_*1*.*1*_ *+qDTY*_*4*.*1*_	5
MABC 6	6 (S)	7 (S)	4 ± 0.4 (MR)	4 ± 0.5 (MR)	R	*Gm8 +Xa21 +qDTY*_*1*.*1*_ *+qDTY*_*2*.*2*_	4
MABC 7	6 (S)	6 (S)	3 ± 0.4 (R)	2 ± 0.4 (R)	R	*Gm8 +Xa21 +qDTY*_*1*.*1*_ *+qDTY*_*4*.*1*_	4
MABC 8	5 (MR)	5 (MR)	10 ± 0.7 (S)	12 ± 0.6 (S)	R	*Gm8 +Xa21 +qDTY*_*1*.*1*_	3
MABC 9	1 (R)	1 (R)	2 ± 0.4 (R)	2 ± 0.2 (R)	R	*Gm8 +Pi9 +Xa21 +qDTY*_*1*.*1*_ *+qDTY*_*2*.*2*_ *+qDTY*_*4*.*1*_	6
MABC 10	7 (S)	7 (S)	2 ± 0.3 (R)	1 ± 0.3 (R)	R	*Gm8 +Xa21* +*qDTY*_*1*.*1*_ +*qDTY*_*2*.*2*_ *qDTY*_*4*.*1*_	5
MABC 11	5 (MR)	5 (MR)	12 ± 0.4 (S)	10 ± 0.7 (S)	R	*Gm8 +qDTY*_*1*.*1*_ *+qDTY*_*2*.*2*_ *+qDTY*_*4*.*1*_	4
MABC 12	1 (R)	1 (R)	2 ± 0.5 (R)	2 ± 0.4 (R)	R	*Gm8 +Pi9 +Xa21 +qDTY*_*1*.*1*_ *+qDTY*_*2*.*2*_	5
Naveen	7 (S)	7 (S)	13 ± 0.5 (S)	14.5 ± 0.4 (S)	S	-	-

Blast Resistant Check- IRBL9 (2 ± 0.2), TETEP (0 cm); Blast Susceptible Check- HR12 (7 ± 0.4); BLB Resistant Check- IRBB60 (0.5 cm); BLB Susceptible Check- TN1 (19 ± 0.8); Gall midge resistant check–Aganni (0 galls); Gall midge susceptible check–TN1 (100 galls).

S-Susceptible; R-Resistance; MR-Moderate resistance.

* Screened at IIRR, Hyderabad GM glass house screening facility using Biotype 1 during WS2018

Blast (SES, IRRI 2013); 0–2 (Resistant), 3–4 (Moderately Resistant), 5–9 (Susceptible)

BLB lesion length Chen., et al. 2000 R (up to 3 cm), MR (> 3–6 cm), MS (> 6–9 cm), S (> 9 cm)

Gall Midge (SES, IRRI 2002); 0, 1, 3 Resistant and 5, 7, 9 Susceptible.

Likewise for BB, the donor IRBB60 showed complete resistance with lesion length of 0.5 cm and the recurrent parent Naveen showed susceptibility with lesion length more than 19 cm. Among the 8 MAFB lines tested, 4 lines (MAFB 5, MAFB 6, MAFB 7 and MAFB 8) showed high resistance (lesion length <3cm) and 2 lines (MAFB 1 and MAFB 2) showed moderate resistance (lesion length >3 to <6cm) in both the seasons of WS2018 and WS2019. **(Table 1)**. Out of 12 MABC lines, 7 lines (MABC 1, MABC 2, MABC 3, MABC 7, MABC 9, MABC 10 and MABC 12) showed high resistance (lesion length <3cm), 2 lines (MABC 4 and MABC 6) showed moderate resistance (lesion length >3 to <6cm) **(Table 2, Fig 2B)**. All the resistance and moderate resistance lines of MAFB and MABC lines found to posses *Xa21* gene **(Tables 1 and 2)**.

All the above MAFB and MABC gene/QTL pyramided lines were screened against gall midge biotype 1 in green house at ICAR-IIRR, Hyderabad during WS2018. The donor parent, Aganni showed 100% resistance while the recurrent parent Naveen and susceptible check TN1 showed high level of susceptibility with more than 70% plants with incidents of galls. Out of the 8 MAFB lines, five lines (MAFB 1, MAFB 3, MAFB 4, MAFB 5 and MAFB 6) found to posses *Gm8* gene and showed resistance with less than 8% of gall formation **(Table 1)**. All the 12 MABC lines found to posses *Gm8* gene and showed high level of resistance **(Table 2)**.

### Agronomic performance and drought tolerance of marker-assisted breeding lines

Drought tolerance studies were conducted during WS2018 at two test fields (IRRI-SAH, Hyderabad and ICAR-IIRR, Hyderabad). Severe drought stress was imposed and the lines were evaluated for their phenotypic expression in terms of days to 50% flowering (DTF), plant height (PH), productive tiller number (PT), Panicle length (PL), and grain yield (Kg/ha) under both control and drought stress conditions. Two drought donors (IR 87707-445-B-B and IR 96321-1447-561-B-1), two susceptible checks (MTU1010 and IR64) and one tolerant check (DRR Dhan44) were used in this study. In MAFB lines, all the traits showed significant variation in drought stress compared to control. The mean DTF varied from 87 to 99 days in control trial and 68 to 96 days under drought stress trial, while the parental DTF is 95 days in control and 87 days under drought stress. Whereas the PH, PT, PL and TGW significantly decreased under drought stress compared to control **(Table 3)**. In control conditions, MAFB lines showed lower GY compared to the parent Naveen. The mean GY of the Naveen under control was recorded as 6253 kg ha^-1^ and the mean GY of the pyramided lines were ranged from 1868 to 4370 kg ha^-1^. Meanwhile under drought stress, the mean GY of Naveen was 1267 kg ha^-1^ and the GY of the MAFB lines was ranged from 855 to 2406 kg ha^-1^. Three lines {MAFB 3 (2008 kg ha^-1^), MAFB 4 (1982 kg ha^-1^) and MAFB 7 (2406 kg ha^-1^)} showed higher GY under drought stress compared to recurrent parent Naveen and also both drought donors {IR 87707-445-B-B (1605 kg ha^-1^) and IR96321-1447-561-B-1 (1934 kg ha^-1^)} and tolerant check (DRR Dhan44 (1902 kg ha^-1^)) **(Table 3)**.

**Table 3:** Agronomic and yield performance of pyramided lines developed through the MAFB approach.

Gene/QTL pyramids	DFF (days)		PH (cm)		PT		PL (cm)		Yield (Kg/ha)*	
	NS	RS	NS	RS	NS	RS	NS	RS	NS	RS
MAFB 1	99	96	76	66	15	11	23	21	4370	1189
MAFB 2	90	79	67	62	14	9	21	19	1868	1292
MAFB 3	88	71	64	50	11	11	19	19	2591	2008
MAFB 4	89	86	77	74	14	10	21	19	2919	1982
MAFB 5	87	70	75	59	12	12	22	19	3449	1264
MAFB 6	92	88	50	47	12	11	19	18	2582	1573
MAFB 7	91	68	56	49	16	12	20	16	3409	2406
MAFB 8	98	85	74	63	15	11	20	19	4265	855
Naveen	95	87	80	69	16	11	20	19	6253	1267
IR 87707-445-B-B	90	110	69	66	16	11	21	20	5583	1622
IR96321-1447-561-B-1	114	95	59	57	15	13	21	18	4946	1934
DRR Dhan44	93	99	74	65	11	11	21	20	5913	1902
MTU1010	92	115	71	68	13	10	22	19	6293	1160
IR64	96	107	65	60	14	10	19	20	5743	1287
Trial mean	93.70	89.43	68.29	60.95	13.74	10.77	20.59	18.80	4299.01	1552.91
F value	2.79*	2.58(NS)	3.50*	6.41**	2.87*	0.67(NS)	1.02(NS)	1.22(NS)	80.23**	7.51**
SED	5.82	13.06	6.56	4.47	1.52	1.78	1.62	1.66	238.86	224.87
Trial heritability	0.68	0.63	0.73	0.86	0.69	0	0.03	0.22	0.99	0.93

DFF—days to 50% flowering (days); PH—plant height (cm); PT–productive tillers; PL—panicle length (cm); Yield (Kg/ha)–grain yield kilo gram per hectare; NS non-stress, RS reproductive stage drought stress; SED–Standard error of difference.

Data represents mean of two trials, one at IRRI-SAH, Patancheru and two at ICAR-IIRR, Rajendranagar.

Compared to MAFB lines, MABC lines were performed very well under drought stress. The mean DTF of MABC lines increased under drought stress (96 to 136 days) compared to control (92 to 101 days). Whereas, the other four traits (PH, PT, PL and TGW) showed significant decrease in mean values **(Table 4)**. Under control conditions, the mean GY of the MABC lines were ranged from 3823 to 8283 kg ha^-1^. Meanwhile under drought stress, the mean GY of the MABC lines ranged from 1364 to 3675 kg ha^-1^. Among all the MABC lines, five lines {MABC 4 (3675 kg ha^-1^), MABC 6 (2706 kg ha^-1^), MABC 7 (3370 kg ha^-1^) MABC 11 (2598 kg ha^-1^) and MABC12 (2681 kg ha^-1^)} showed significant yield increase compared to recurrent parent Naveen (1267 kg ha^-1^), drought donors {IR 87707-445-B-B (1605 kg ha^-1^) and IR96321-1447-561-B-1 (1934 kg ha^-1^)} and tolerant check {DRR Dhan44 (1902 kg ha^-1^)} **(Table 4)**. In control, six lines yielded higher grain yield than Naveen. Overall 2 lines (MABC 6 and MABC 7) showed higher GY than Naveen, donors and check under both control and drought stress conditions.

**Table 4:** Agronomic and yield performance of selected pyramided lines developed through the MABC approach.

Gene/QTL pyramids	DFF (days)		PH (cm)		PT		PL (cm)		Yield (Kg/ha)*	
	NS	RS	NS	RS	NS	RS	NS	RS	NS	RS
MABC 1	94	136	76	69	14	10	20	18	6382	1561
MABC 2	96	114	75	68	14	9	20	21	5959	1364
MABC 3	95	98	79	72	14	11	21	21	5134	1907
MABC 4	95	96	67	66	13	10	18	18	3823	3675
MABC 5	98	99	70	59	15	11	20	18	8283	1885
MABC 6	92	103	75	67	12	12	20	19	6859	2706
MABC 7	101	125	79	77	14	11	22	23	8187	3370
MABC 8	96	121	88	73	17	11	23	21	7700	1598
MABC 9	96	119	64	56	12	14	20	17	3864	1498
MABC 10	93	114	76	67	11	11	21	19	7040	1496
MABC 11	93	109	81	66	13	13	19	20	5218	2598
MABC 12	98	120	83	71	15	11	20	20	6052	2681
Naveen	94	87	80	69	16	11	20	19	6075	1267
IR 87707-445-B-B	90	110	69	66	16	11	21	20	5583	1605
IR96321-1447-561-B-1	114	95	59	57	15	13	21	18	4946	1934
DRR Dhan 44	93	99	74	65	11	11	21	20	5913	1902
MTU 1010	92	115	71	68	13	10	22	19	6293	1160
IR64	96	107	65	60	14	10	19	20	5743	1287
Trial mean	95.86	109.19	73.93	66.35	13.69	10.94	20.42	19.45	6058.55	1971.82
F value	1.30(NS)	1.77(NS)	5.39**	2.97*	2.23(NS)	0.74(NS)	0.66(NS)	0.90(NS)	8.02**	4.78**
SED	6.57	13.34	4.51	4.44	1.71	1.87	1.84	1.99	628.20	475.69
Trial heritability (H)	0.26	0.44	0.83	0.68	0.59	0	0	0	0.92	0.82

DFF—days to 50% flowering (days); PH—plant height (cm); PT–productive tillers; PL—panicle length (cm); Yield (Kg/ha)–grain yield kilo gram per hectare; NS non-stress, RS reproductive stage drought stress; SED–Standard error of difference.

#Data represents mean of two trials, one at IRRI-SAH, Patancheru, Hyderabad and two at ICAR-IIRR, Rajendranagar, Hyderabad.

### Grain quality analysis of marker-assisted breeding lines

The grain quality data (WS2018) of 8 MAFB lines and 12 MABC lines under control and drought stress are presented in Tables 5 and 6. Kernel length (KL) of MAFB lines ranged from 5.8 to 7.1 mm under control and 5.2 to 6.2 mm under drought (Table 5), whereas the KL of MABC lines was ranged from 5.6 to 6.5 mm under control and 5.3 to 6.2 mm under drought conditions (Table 6) in comparison to 6.2 and 6.1 mm of the recurrent parent (Naveen) in both the conditions. Kernel breadth (KB) of MAFB lines ranges from 2.1 to 2.6 mm under control and 1.9 to 2.2 mm under drought stress (Table 5), whereas the KB of MABC lines was ranged from 2.1 to 2.6 mm under control and 1.9 to 2.4 mm under drought conditions (Table 6) in comparison to 2.4 and 2.2 mm of the Naveen in both the conditions respectively. Based on the L/B ratio, majority of the MAFB and MABC lines were categorized under medium bold (MB) grain type, which is similar to the Naveen.

**Table 5:** Grain quality characteristics of MAFB lines under control and drought conditions during WS2018.

Gene/QTL pyramids	KL (mm)		KB (mm)		LBR (mm)		GC(mm)		AC (%)	
	NS	RS	NS	RS	NS	RS	NS	RS	NS	RS
MAFB 1	6.4 (m)	5.8 (m)	2.1	2.0	3.0 (m)	2.9 (m)	65.4	45.7	22.3 (I)	20.0 (I)
MAFB 2	5.8 (m)	5.2 (s)	2.2	2.2	2.7 (m)	2.4 (m)	82.5	46.8	25.4 (H)	24.0 (I)
MAFB 3	6.4 (m)	6.1 (m)	2.2	2.2	2.9 (m)	2.8 (m)	65.0	47.5	26.5 (H)	25.0 (I)
MAFB 4	7.1 (l)	6.2 (m)	2.3	2.0	3.0 (m)	3.1 (s)	45.2	39.0	24.0 (I)	23.0 (I)
MAFB 5	6.1 (m)	6.0 (m)	2.2	2.4	2.8 (m)	2.5 (m)	85.0	44.1	26 (H)	24.7 (I)
MAFB 6	6.4 (m)	5.8 (m)	2.3	2.0	2.8 (m)	2.9 (m)	82.5	82.3	22.5 (I)	21.0 (I)
MAFB 7	6.1 (m)	6.0 (m)	2.6	2.2	2.4 (m)	2.7 (m)	62.5	37.7	23.4 (I)	22.9 (I)
MAFB 8	7.0 (l)	6.1 (m)	1.9	1.9	3.4 (s)	3.2 (s)	57.5	52.5	23.3 (I)	22.3 (I)
Naveen	6.2 (m)	6.1 (m)	2.4	2.2	2.5 (m)	2.8 (m)	87.5	54.5	25.7 (H)	22.0 (I)
Trial mean	6.37	5.95	2.25	2.13	2.87	2.82	70.34	50.01	24.34	22.77
F value	82.87**	76.46**	104.17**	41.31**	79.71**	38.75**	14.30**	11.93**	1.90*	1.01 (NS)
SED	0.07	0.05	0.03	0.03	0.06	0.06	5.48	5.44	1.62	2.32
Trial heritability (H)	0.99	0.99	0.99	0.98	0.99	0.97	0.93	0.92	0.48	0

KL–Kernel length; KB–Kernel breadth; LBR—length-breadth ratio; GC–gel consistency; AC amylose content, (L–low amylase content; I—intermediate amylose content (20–25%); H—high amylose content (> 25%)); SED–Standard error of difference.

Kernel length (scale): 1.Extra long (el > 7.5 mm), 3.long (l—6.6–7.5 mm), 5.medium (m—5.51–6.6 mm), 7. short (s—5.5mm or < 5.5 mm) (SES, IRRI 2013)

Grain type classification (combination of KL and LBR), Length-breadth ratio (scale): 1. slender (> 3), 3. medium (2.1–3), 5. bold (1.1–2), 7. round (< 1.1) (SES, IRRI 2013)

**Table 6:** Grain quality characteristics of MABC lines under control and drought conditions during WS2018.

Gene/QTL pyramids	KL (mm)		KB (mm)		LBR (mm)		GC(mm)		AC (%)	
	NS	RS	NS	RS	NS	RS	NS	RS	NS	RS
MABC 1	6.5 (m)	6.2 (m)	2.6	2.4	2.5 (m)	2.6 (m)	82.5	79.8	26.9 (H)	22.1 (I)
MABC 2	6.2 (m)	5.7 (m)	2.2	1.9	2.9 (m)	3.0 (m)	75.3	58.2	23.9 (I)	17.7 (L)
MABC 3	5.9 (m)	5.6 (m)	2.3	2.1	2.6 (m)	2.7 (m)	75.0	90.5	25.3 (H)	23.8 (I)
MABC 4	5.9 (m)	5.8 (m)	2.3	2.2	2.6 (m)	2.6 (m)	83.3	37.8	23.7 (I)	22.3 (I)
MABC 5	5.9 (m)	5.3 (s)	2.2	2.0	2.7 (m)	2.7 (m)	72.5	42.3	26.3 (H)	25.1 (H)
MABC 6	6.1 (m)	6.1 (m)	2.2	2.2	2.8 (m)	2.9 (m)	60.3	49.6	26.2 (H)	22.9 (I)
MABC 7	6.1 (m)	5.4 (s)	2.2	2.0	2.8 (m)	2.8 (m)	55.3	60.6	25.1 (H)	23.8 (I)
MABC 8	6.1 (m)	6.0 (m)	2.2	2.0	2.8 (m)	3.0 (m)	70.3	75.3	25.1 (H)	24.3 (I)
MABC 9	6.1 (m)	5.8 (m)	2.2	2.0	2.8 (m)	2.9 (m)	67.4	85.6	23.3 (I)	23.0 (I)
MABC 10	6.0 (m)	5.4 (s)	2.1	2.1	3.0 (m)	2.5 (m)	62.4	44.9	24.9 (I)	23.3 (I)
MABC 11	5.6 (m)	5.4 (s)	2.2	2.0	2.6 (m)	2.7 (m)	86.3	46.6	26.9 (H)	25.8 (H)
MABC 12	6.1 (m)	5.8 (m)	2.1	2.0	3.0 (m)	2.9 (m)	57.5	76.1	26.3 (H)	25.7 (H)
Naveen	6.2 (m)	6.1 (m)	2.4	2.2	2.5 (m)	2.8 (m)	87.5	54.5	25.7 (H)	22.0 (I)
Trial mean	6.05	5.74	2.25	2.09	2.72	2.77	71.99	61.67	25.36	23.19
F value	15.20**	139.79**	20.79**	12.049**	11.46**	7.061**	5.466**	16.82**	0.9405 (NS)	3.414*
SED	0.08	0.04	0.04	0.05	0.07	0.08	6.64	6.13	1.73	1.56
Trial heritability (H)	0.93	0.99	0.95	0.92	0.91	0.86	0.82	0.94	0	0.71

KL–Kernel length; KB–Kernel breadth; LBR—length-breadth ratio; GC–gel consistency; AC amylose content, (L–low amylase content; I—intermediate amylose content (20–25%); H—high amylose content (> 25%)); SED–Standard error of difference.

Kernel length (scale): 1.Extra long (el > 7.5 mm), 3.long (l—6.6–7.5 mm), 5.medium (m—5.51–6.6 mm), 7. short (s—5.5mm or < 5.5 mm) (SES, IRRI 2013)

Grain type classification (combination of KL and LBR), Length-breadth ratio (scale): 1. slender (> 3), 3. medium (2.1–3), 5. bold (1.1–2), 7. round (< 1.1) (SES, IRRI 2013).

Apart from KL, KB and L/B ratio, gel consistency (GC) and amylose content (AC) of all the marker-assisted breeding lines were recorded under both control and stress conditions. Significant differences were observed among these traits in between control and drought. GC varied significantly among the lines. In MAFB lines, it was ranged from 45 to 85 under control and 38 to 82 under stress (Table 5) where as in MABC lines it ranged from 55 to 86 under control and 38 to 90 under stress (Table 6). The GC of Naveen was 87.5 under control and 54.5 under stress. Amylose content (AC) of majority of the marker-assisted breeding lines under control were of intermediate to high type (AC ranged from 22.3 to 26.5% in MAFB lines and 23.3 to 26.9% in MABC lines), similar to the recurrent parent (25.7%). Under drought stress, AC is ranged from 20 to 25% in MAFB lines and 17.7 to 25.8% in MABC lines whereas in Naveen it was recorded as 22%. These results indicate that drought has some effect on grain quality characteristics.

## Discussion

Naveen is a mid-early duration, high yielding, medium bold rice variety released in the year 2006 and adopted by the Indian farmers in the regions of Odisha and Tripura. Improving Naveen for blast, BB, GM resistance and drought tolerance will invariably help the farmers to reduce the use of pesticides thereby reducing the production cost and also to sustain under water deficit conditions. In the present study, we followed two marker-assisted breeding approaches, namely marker-assisted forward breeding (MAFB) and marker assisted backcrossing (MABC) to combine three biotic stress resistance genes and three drought tolerance QTLs into Naveen (Fig 1). Majority of the high yielding, consumer acceptable rice varieties are being rejected for cultivation by farmers due to lack of biotic and abiotic stress resistance/tolerance or poor grain quality traits even if they produce high yield. This can be overcome by rapid and precise introgression of multiple genes/QTLs which provides resistance/tolerance to multiple biotic and abiotic stresses. Tagging, mapping and cloning of various genes which possess resistance against biotic stresses such as blast [52], bacterial blight (BB) [53], and gall midge (GM) [22] facilitated the development of reliable DNA markers linked to those genes and gave boost to marker assisted breeding for multiple stress resistance. In the past few years, several popular elite high yielding rice varieties were developed by incorporating these (blast, BB and GM) resistance genes through marker assisted selection (MAS) involving both foreground and background selection [28, 54–60]. Not only the biotic stresses, significant success has been achieved in pyramiding QTLs associated with tolerance to abiotic stresses such as drought tolerance [50, 61], submergence tolerance [62, 63] and salinity tolerance [64, 65] through MAS. These reports provide evidence for the usefulness of MAS for development of biotic as well as abiotic stress resistant/tolerant rice varieties.

In recent years, marker-assisted breeding has been advanced from introgression of single/multiple genes conferring resistance for single disease in single genotype to multiple genes conferring resistance to multiple diseases in single genotype. There are reports in rice where multiple genes conferring resistance to two different biotic stresses were introgressed into the background of popular rice varieties for combined resistance to target pests such as BB and blight [20, 66] or BB and GM [27]. Attempts have been also made for the introgression of multiple biotic (blast, BB and GM) and abiotic stress (salinity and submergence) resistance/tolerance genes/QTLs together into improved rice varieties (improved Lalat and improved Tapaswini) and succeeded in achieving multiple stress tolerance [28, 61] in the resultant pyramided lines. But there is no previous report on successful introgression of biotic stress resistance genes together with drought tolerant QTLs. Moreover, the reported studies followed marker assisted backcross breeding (MABC) approach for pyramiding additional stress resistant/tolerant genes/QTLs into already improved rice varieties with existing resistance genes for a biotic stress [28, 60]. Although transferring multiple genes is feasible with MABC, the major constraint associated with the multiple introgressions is decline in yield potential of pyramided lines. In both the previous studies of the introgression of multiple genes/QTLs, researchers reported significant yield loss in the pyramided lines [28, 60].

A combined approach of MAFB with MABC breeding was reported by Kumar *et al*. [67] which helped in recovery of all the desirable traits along with target traits and also grain yield advantage in multiple gene introgressions in wheat. In the present study, we also followed a combined approach of MAFB and MABC to bring all the desired genes/QTLs into Naveen. Initially through MAFB approach all the genes and QTLs were brought into the common genetic background. Once after identifying the lines with all genes/QTLs combinations (IC_3_F_3_), some of ILs were forwarded and developed as RILs (MAFB) and few were backcrossed with Naveen to develop BILs (MABC) for retaining higher percent of the recurrent parent genome. Stringent field based selections and foreground screening with markers was followed at each generation of MAFBs and MABCs and the identified genes/QTLs combinations were stabilized only at F_7_ generation in case of MAFBs and BC_1_F_4_ generation in case of MABCs. Marker-assisted breeding lines which showed same genes/QTLs combinations in homozygous conditions (F_7_, and BC_1_F_4_) were selected and evaluated for their biotic stress resistance at both seasons (WS2018 and WS2019) and agronomic performance and drought stress tolerance at two independent field experiments (IRRI-SAH, Hyderabad and ICAR-IIRR, Hyderabad). Compared to MAFB lines, lines developed through combined approach of MAFB and MABC showed higher yield under both drought and control conditions and also biotic stress resistance. In control conditions, none of the MAFB lines showed higher yield compared to recurrent parent Naveen. The mean GY of the Naveen under control was recorded as 6253 kg ha-1 and the mean GY of the pyramided lines were ranged from 1868 to 4370 kg ha-1. Meanwhile under drought stress, the mean GY of Naveen was 1267 kg ha-1 and the GY of the MAFB lines was ranged from 855 to 2406 kg ha-1 respectively. Three MAFB lines (MAFB 3, MAFB 4 and MAFB 7) showed higher GY (36–47%) under drought stress compared to recurrent parent Naveen. In case of MABC lines, significant higher yields were observed in both control and stress conditions compared to Naveen. Under control conditions, the mean GY of the MABC lines were ranged from 3823 to 8283 kg ha-1. Meanwhile under drought stress, the mean GY of the MABC lines ranged from 1364 to 3675 kg ha-1. Out of 12 MABC lines, six lines (MABC 1, 5, 6, 7, 8 and 10) showed significant higher grain yield (5–27%) than Naveen in control conditions and five lines (MABC 4, 6, 7, 11 and 12) showed significant higher yield (51–66%) than Naveen under drought stress conditions. The superior performance of MABC lines in comparison with MAFB lines may have resulted from breakage of undesirable linkages or loss of negative interactions as it went through one round of backcrossing and advancement. There are various reports available on the interaction between introgressed genes and homolog reporter sequences in transgenic rice but very few for MAS. Sandhu *et al*., [50] has studied the allelic profile of MAS pyramided lines (PLs) having same *qDTY* combination but with variable phenotypic expression under drought and concluded that, interactions among favorable allele, increase in positive alleles frequency over negative allele frequency, haplotype mediated controlling of the traits, and variable expression of genomic regions under differential conditions may be responsible factors. Similarly, Yadav *et al*., [68] also reported that effect of epistatic interactions between QTLs and genetic background, interactions between background markers loci of different chromosomes and QTL-QTL interactions may play a major role. Among the 8 MAFB lines, two lines (MAFB 1 and MAFB 6) showed MR to compete R to all 3 biotic stresses (blast, BB and GM). Moreover, except three lines (MAFB 1, MAFB 5 and MAFB 8) all other five lines showed GY advantage over RP Naveen under drought stress. Among these seven lines, three lines (MAFB 3, 4 and 7) not only showed higher yields than Naveen but also over drought tolerant donors and checks used in the study. Whereas in MABC lines, three lines (MABC 2, MABC 9 and MABC 12) showed MR to compete R to all 3 biotic stresses (blast, BB and GM). Coming to drought stress, all lines showed higher yields compared to Naveen and five lines (MABC 4, 6, 7, 11 and 12) lines showed higher yields compared drought tolerant donors and checks used in the study. The 3 MAFBs (MAFB 3, 4 and 7) and five MABCs (MABC 4, 6, 7, 11 and 12) were considered as high priority lines due to their higher GY advantage under drought stress. These results signify the importance of the combined approach of MAFB with MABC breeding.

The major problem associated with multiple gene/QTL introgressions is failure of the phenotypic expression of some of the genes/QTLs in the pyramided lines. Among all the lines, only one line (MABC 9) showed the presence of all 6 genes/QTL combinations. The non-recovery of some of the gene/QTL combinations is attributed to genotypic specificity by some of the previous MAS breeding studies [69]. Moreover, some of the pyramided lines were found to show susceptibility to particular stress, even though respective gene/QTL was identified in PCR analysis. In general, during multiple gene/QTL pyramiding programs, it is expected that introgressed genes/QTLs act together and exhibit combined resistance/tolerance. But the interactions occurring among the introgressed genes/QTLs are not well studied. There is a possibility of significant antagonistic as well as synergistic interactions among the introgressed genes/QTLs in the genome which results in either suppressing or activation of the function of other genes [2, 70]. In our study, some of the marker-assisted breeding line showed susceptibility to biotic stress (*Xa21* in MABC 5 and MABC 8) and drought stress (MAFB 1, MAFB 8) though the respective gene/QTL was present genotypically. This may be due to the antagonistic interactions occurring among the gene/QTL combinations present in those lines. Similar results were reported by Divya *et al*., [19] where they have evaluated a set of ten pyramided lines against blast, BB and GM diseases and found that that their expression was not in accordance with the PCR identification of the respective genes. Especially the lines which are confirmed genotypically for the presence of resistance genes for gall midge (*Gm1* and *Gm4*) and blast (*Pi2* or *Pi54*) are found to show susceptibility to respective stresses. Das *et al*., [59] also reported the occurrence of negative interactions among disease resistance genes introgressed in rice. Further, some of the marker-assisted breeding lines showed resistance to biotic stress though the respective gene was not present genotypically. This may be due to the synergistic interactions occurring among the gene/QTL combinations present in those lines. The lines MAFB 4, MAFB 6 and MABC 2, MABC 8, MABC 11 showed moderate resistance to blast though the *Pi9* gene is not identified in these lines. This could be attributed to background interaction of genes/QTLs in those ILs which may lead to positive epistatic interaction in the genome which is reported by earlier few studies [50, 56, and 68]. Moreover, some of the lines with same gene/QTL combinations (MAFB 5 and 6; MABC 1, 2 and 3; MABC 4, 8; MABC 5, 7) showed differential phenotypic response in the present study. However, an in-depth understanding on the gene-gene interactions or gene-QTL interactions is needed to make further conclusions.

In the present study, a systematic pyramiding approach was followed involving both MAFB and MABC breeding strategy which resulted in development of lines similar to recurrent parent morphologically but with different gene/QTL combinations. In rice, this is the first time we are reporting the combined approach of MAFB and MABC which yielded positive results for introgression of multiple genes/QTL. Though it is possible to transfer multiple genes through MAFB, combining backcross breeding helped us in development of lines with Naveen characteristics along with target traits. Moreover it also helped in minimizing the donor type segments. The large number of BC_1_F_1_s and presence of high variance in succeeding generations, indicate a wider range of frequency distribution in the BC_1_F_1_ suggesting the feasibility of practicing selections in the BC_1_F_1_ generation [56]. Despite of using six different parents, stringent field based phenotypic selections based on different agro-morphological characters at each generation helped in identification of lines with superior performance over recurrent parent (Naveen) in terms of quality and stress tolerance. This study is the practical example of a combination of MAFB and MABC approaches which clearly demonstrated the superiority over conventional backcrossing with superior agronomic performance. The improved Naveen lines that tolerate both biotic and abiotic stress can help in understanding the epistatic interactions occurring among the introgressed genes/QTL and their interaction with genetic background. Moreover, it also serves as valuable resource for crop improvement programs targeted for improving multiple biotic/abiotic stress resistance/tolerance and agronomic traits. The success of the introgression of multiple genes/QTLs into a single background through marker-assisted breeding will further pave way for the plant breeders to introgress more number of desirable traits to develop a plant type with combination of genes/QTLs through non-transgenic approaches. These improved Naveen lines can be tested in multi-locations across the country through All India Coordinated Rice Improvement Program (AICRIP-Rice) for their respective high yield and biotic and abiotic stress resistance/tolerance. After 3 years of rigorous testing, these lines can be released to the farmers in the respective areas. Naveen was primarily released and adopted by the farmers in the states Odisha and Tripura where it can be replaced by improved Naveen lines.

## Conclusion

In the present study, an Indian elite rice variety Naveen was improved for biotic and abiotic stress resistance/tolerance by pyramiding biotic stress resistance genes for blast (*Pi9*), BB (*Xa21*) and GM (*Gm8*) and three drought tolerant QTLs (*qDTY1*.*1*, *qDTY2*.*2* and *qDTY4*.*1*). Marker assisted forward and backcross breeding approach was followed to introgress multiple gene/QTLs to develop introgression lines without any yield penalty. Through stringent phenotyping in field based on agro-morphological characteristics and foreground selection with tightly linked markers at each generations, eight lines through MAFB approach and 12 lines through combined MAFB and MABC approach were identified with similar characteristics to recurrent parent Naveen and with increased level of tolerance/resistance to drought and other three biotic stresses. The success of this study clearly exemplifies the efficacy of the combined approach of MAFB and MABC strategy in pyramiding multiple biotic and abiotic stress resistance/tolerance genes/QTLs into an elite genetic background, obtain introgression lines with higher or similar yield of the recurrent parent under control. Further, the marker-assisted breeding lines will be screened in multi-location trials under the All India Coordinated Research Projects-Rice (AICRP-Rice) for their possible release for the benefit of rice farmers. The developed marker-assisted breeding lines can also be used as novel breeding source in crossing programs for improvement of number of elite rice varieties.

## Supporting information

S1 Table List of donors and markers used for foreground selection of blast, BB, Gall midge resistance genes and drought tolerance QTLs, their chromosome location, and primer sequence. (DOCX)
