# Supplementary material for: Marker-assisted forward and backcross breeding for improvement of elite Indian rice variety Naveen for multiple biotic and abiotic stress tolerance
Source: PLoS One. 2021 Sep 2;16(9):e0256721. doi: 10.1371/journal.pone.0256721 (PMC8412243; doi:10.1371/journal.pone.0256721)
Supplement: S1 Table — (DOCX) [file pone.0256721.s001.docx]

**S1 Table.** List of donors and markers used for foreground selection of blast, BB, Gall midge resistance genes and drought tolerance QTLs, their chromosome location, and primer sequence

| **S.no** | **Gene/QTL** | **Trait** | **Donors** | **Gene based/Linked Markers** | **Primer sequence** | **Ch No.** | **References** |
| --- | --- | --- | --- | --- | --- | --- | --- |
| 1 | *Pi9* | Blast | IRBL9 | *Pi9STS2* | GGAGAATCCGAGAAGCAGAACTA ACAACTTGAACTAGTACGGCGATG | 6 | Qu *et al.,* (2006) |
| 2 | *Xa21* | BLB | IRBB60 | *pTA248* | AGACGCGGAAGGGTGGTTCCCGGA AGACGCGGTAATCGAAAGATGAAA | 11 | Ronald *et al.*, (1992) |
| 3 | *Gm8* | Gall midge | Aganni | *PRP* | TCATGTTGTGCAGATCAACC AGCCATATGAAAACCACCAA | 8 | Divya *et al.,* (2013) |
| 4 | *qDTY_1_*_._*_1_* | Drought | IR96321-1447-561-B-1 | RM3825 (LFM) | AAAGCCCCCAAAAGCAGTAC GTGAAACTCTGGGGTGTTCG | 1 | Vikram *et al*., (2015) |
|  |  |  |  | RM431 (PM) | TCCTGCGAACTGAAGAGTTG AGAGCAAAACCCTGGTTCAC |  |  |
|  |  |  |  | RM12091 (RFM) | CTGCAAATGCACAGGAATCAGG TCCTCTCGCCTTTCTTTCTCTCC |  |  |
| 5 | *qDTY_2.2_* | Drought | IR87707-445-B | RM154 (LFM) | GACGGTGACGCACTTTATGAACC CGATCTGCGAGAAACCCTCTCC | 2 | Swamy *et al.,* (2013) |
|  |  |  |  | RM279 (PM) | GCGGGAGAGGGATCTCCT GGCTAGGAGTTAACCTCGCG |  |  |
|  |  |  |  | RM555 (RFM) | TTGACATGCGAAATGGAGATGG TTGGATCAGCCAAAGGAGACC |  |  |
| 6 | *qDTY_4_*_._*_1_* | Drought | IR87707-445-B | RM551 (LFM) | CTTACTCCATTGGGCTGGAACC TGTAGGGTGGTAAGAGATCCACTCC | 4 | Swamy *et al.,* (2013) |
|  |  |  |  | RM518 (PM) | CTCTTCACTCACTCACCATGG ATCCATCTGGAGCAAGCAAC |  |  |
|  |  |  |  | RM16367 (RFM) | GTTCCCAACCGGAACAGTACG CCCTTAGGCTGTGTTTGACATCC |  |  |

*LFM-Left flanking marker; PM-Peak marker; RFM-Right flanking marker
